# Sleep

**Published:** 1889-05

**Authors:** 


### SLEEP.

Among the phenomena of life, no other one is more mysterious, or farther beyond our comprehension than sleep. In fact, no greater mystery of human life is anywhere presented to our minds than this.

We see man at one moment in full active expression of vigor and strength, capable of grasping a wide range of material and philosophical truths—the next he lies before us unconscious and motionless, save the gentle and involuntary motion of the heart and lungs which continue their unceasing labor.

If there is on record a phenomenon of life that is shrouded in unknown laws, beyond the possibility of obtaining any clue to its philosophy, it is sleep. As mysterious as the trance appears, after having an opportunity to witness and carefully examine a number of cases in this condition, I was able to arrive at some definite conclusions pertaining to but sleep—as it appears to me, is to human conception, miraculous in every sense of this much abused word.

There is no other law which demands obedience, in order to maintain physical existence and perfection that is more imperative, than the hidden one that governs sleep.

Without sleep we cannot be nourished, and one will starve to death as quickly for want of it as from lack of food. It is during sleep that the vital forces, replenish, with new material, the continually wasting tissues of the body.

While we sleep, then, is the redeeming time of God to His children. In this condition He puts into silent repose all their thoughtless, rebellious nature, and accomplishes His work of redemption by replenishing and repairing the exhaustion, waste and injuries which they have sustained from exercise, and oft-times abuse, during voluntary life.

If we are strong, vigorous, and endowed with an abundant stock of vitality, we may succeed in digesting food when asleep, but it is not possible for assimilation—the building up process—to take place when we are awake. If digestion and assimilation are both attempted during sleep, the vital forces will be divided, and neither process will be accomplished so perfectly as if carried on separately.

Those who have realized the necessity of paying strict attention to their physical condition, need to give earnest heed to this subject in order to obtain the best results from their sleep.

It is better to take this repose when all is quiet, than amid noisy surroundings. Sleep obtained before midnight is of much more value than that gotten later in the night or during the day. Yet sleeping at any time is better than not at all.

The best position of the body when asleep is lying on the right side, slightly inclined backward, nearly straight, and with the head toward the north.

There is no time when free and perfect ventilation is so important as when we are asleep. The materials of which the bed is made is also of much importance ; vegetable matter being the best for that purpose.

Animal matter is a poor conductor of electricity, and is not cleanly, metallic beds conduct the electricity too rapidly from the body, and this also disturbs its electric conditions. A bed of straw covered with a light cotton mattress is perhaps the very best.

The amount of sleep required, varies in different persons, and is governed by temperament and occupation. The number of hours given to sleeping should be from five to twelve out of every twenty-four.

The manner of awakening should not be overlooked. One should not be aroused hurriedly, nor is it best to arise in haste.

A clear conscience is necessary to induce sound sleep.—DR. PINKSTAN in the *Journal of Hygeio-Therapy*.

